# Can a key boreal *Calanus* copepod species now complete its life-cycle in the Arctic? Evidence and implications for Arctic food-webs

**DOI:** 10.1007/s13280-021-01667-y

**Published:** 2021-11-29

**Authors:** Geraint A. Tarling, Jennifer J. Freer, Neil S. Banas, Anna Belcher, Mayleen Blackwell, Claudia Castellani, Kathryn B. Cook, Finlo R. Cottier, Malin Daase, Magnus L. Johnson, Kim S. Last, Penelope K. Lindeque, Daniel J. Mayor, Elaine Mitchell, Helen E. Parry, Douglas C. Speirs, Gabriele Stowasser, Marianne Wootton

**Affiliations:** 1 British Antarctic Survey, High Cross, Madingley Rd, Cambridge, CB3 0ET UK; 2 Department of Mathematics and Statistics, University of Strathclyde, Livingstone Tower, 26 Richmond St, Glasgow, G1 1XH UK; 3 University of Franche-Comté, 3 Rue Claude Goudimel, 25000 Besançon, France; 4 Plymouth Marine Laboratory, Prospect Place, West Hoe, Plymouth, PL1 3DH UK; 5 National Oceanography Centre, European Way, Southampton, SO14 3ZH UK; 6 Scottish Association for Marine Science, Dunstaffnage Marine Laboratory, Dunbeg, Oban, Argyll and Bute, PA37 1QA UK; 7 Institute for Arctic and Marine Biology, UiT The Arctic University of Norway, Breivika, 9037 Tromsø, Norway; 8 Department of Biological and Marine Sciences, University of Hull, Hull, HU6 7RX UK; 9 The Laboratory, Citadel Hill, Plymouth, PL1 2PB UK

**Keywords:** Biogeography, Fram Strait, Life-cycle, Ocean warming, Sea-ice loss, Zooplankton

## Abstract

The changing Arctic environment is affecting zooplankton that support its abundant wildlife. We examined how these changes are influencing a key zooplankton species, *Calanus finmarchicus*, principally found in the North Atlantic but expatriated to the Arctic. Close to the ice-edge in the Fram Strait, we identified areas that, since the 1980s, are increasingly favourable to *C. finmarchicus*. Field-sampling revealed part of the population there to be capable of amassing enough reserves to overwinter. Early developmental stages were also present in early summer, suggesting successful local recruitment. This extension to suitable *C. finmarchicus* habitat is most likely facilitated by the long-term retreat of the ice-edge, allowing phytoplankton to bloom earlier and for longer and through higher temperatures increasing copepod developmental rates. The increased capacity for this species to complete its life-cycle and prosper in the Fram Strait can change community structure, with large consequences to regional food-webs.

**Supplementary Information:**

The online version contains supplementary material available at 10.1007/s13280-021-01667-y.

## Introduction

The Arctic is experiencing the strongest warming on the planet and, in recent decades, an unprecedented loss of sea ice (Stroeve and Notz 2018). Arctic warming is not uniform, but amplified in certain regions, such as where there is enhanced inflow of warm Atlantic water into the Eurasian sector of the Arctic Ocean, termed “Atlantification” (Årthun et al. 2012). As these warmer waters further encroach, they bring with them boreal Atlantic species that alter Arctic community structure and affect how food-webs function (Kortsch et al. 2015; Polyakov et al. 2020).

At the base of these food-webs are microscopic zooplankton that principally feed on phytoplankton (primary producers) while themselves being a major prey-source for fish, birds, seals and whales. Within the Arctic and the northern seas, copepods of the genus *Calanus* are one of the most important zooplankton groups, dominating biomass and playing a key role in food webs and biogeochemical cycles (Falk-Petersen et al. 2009). The *Calanus* community in the region consists of three main species. *Calanus glacialis* and *C. hyperboreus* are considered true Arctic species with distributional centres limited mainly to cold, Arctic and Arctic-influenced waters (Falk-Petersen et al. 2009). *Calanus finmarchicus* is smaller and more typically associated with Atlantic water masses but has recently undergone a poleward distributional shift (Wassmann et al. 2020) increasing its contribution to the total *Calanus* community biomass in several Arctic regions (Weydmann et al. 2014; Aarflot et al. 2018; Møller and Nielsen 2020), possibly at the expense of *C. glacialis* and *C. hyperboreus* (Aarflot et al. 2018; Ershova et al. 2021).

**Fig. 1:**
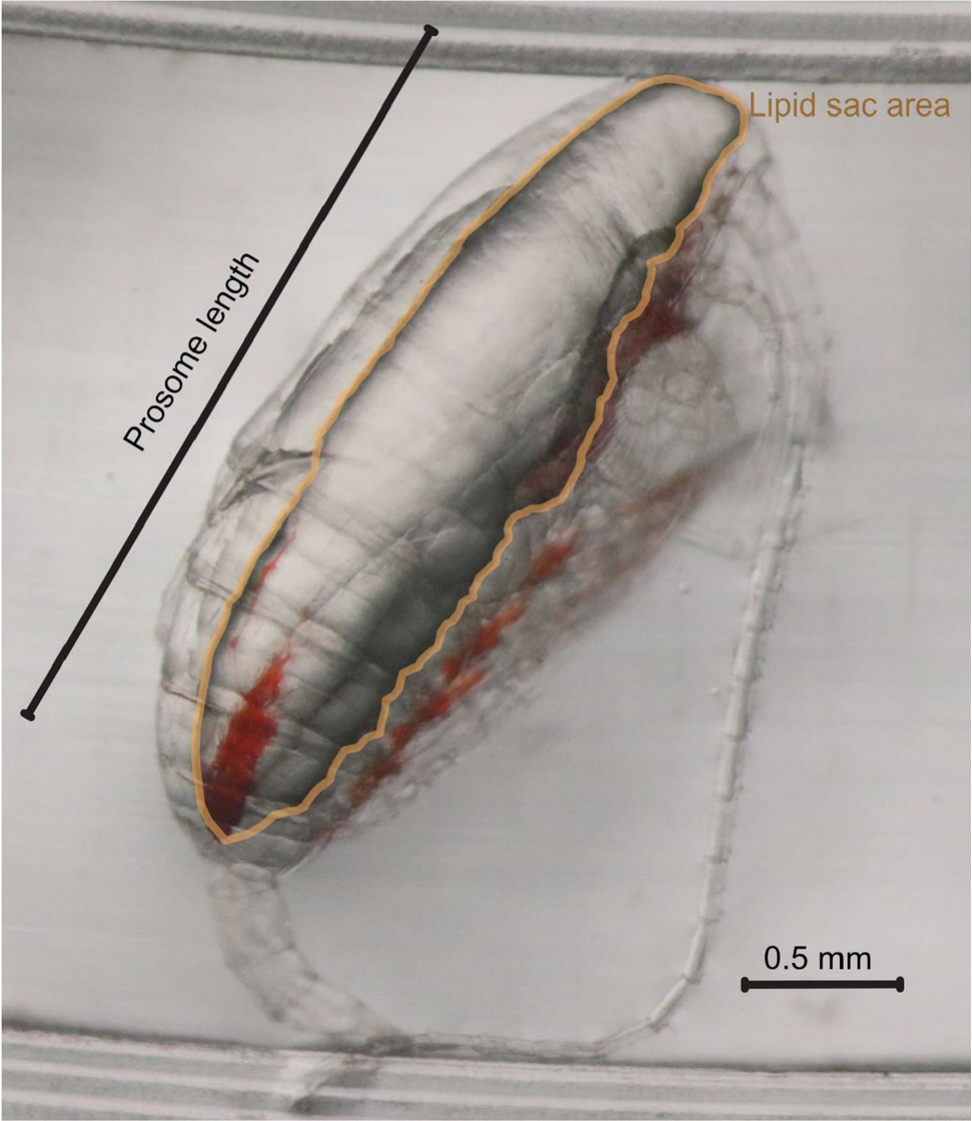
*Calanus finmarchicus* CV taken from deep water layer in Fram Strait during August 2019. The specimen is positioned on a calibrated rimmed Petri dish to facilitate digital biometric analysis. Critical dimensions taken during this analysis also shown

*Calanus* species store energy-rich lipids (principally wax esters, WEs) in a membrane-bound organ, the lipid sac (Fig. 1) that can fill over half of the volume of the prosome (Miller et al. 1998). Lipid sac size increases with developmental stage, reaching its largest size in either the final pre-adult stage (copepodite five, CV) or the adult stage. The life-cycle of *Calanus* in northern waters includes a period of overwintering where late developmental stages (principally CIV or CV) or adult stages outlast the dark winter months in a state of diapause where they descend to depths of between 500 and 2000 m, lower their metabolic rate (Saumweber and Durbin 2006) and become relatively inactive (Hirche 1996). The lipid sacs fuel their residual energy requirements during this time and further facilitate the moult to adulthood, mate-finding and at least some egg production the following spring (Jónasdóttir et al. 2019).

The timing of egg spawning is critical since the phytoplankton blooms that young developmental stages feed upon are often short lived and dependent on the break-up of sea-ice. Spawning that takes place prematurely may result in starvation of the developing larvae, while spawning too late results in insufficient time for the later developmental stages to build up the required reserves to overwinter (Varpe et al. 2007). The added complexity is that the break-up of ice, which constrains the length of the productive season in open water, is spatially and temporally variable (Wassmann 2011; Lind et al. 2018). *Calanus glacialis* can exploit ice-algae which can precede the open-water phytoplankton bloom by 1–2 months (Søreide et al. 2008) which decreases their reliance on seasonal sea-ice break up to facilitate feeding (Daase et al. 2013). Furthermore, both *C. glacialis* and *C. hyperboreus* have multi-year life-cycles that gives them the flexibility to cope with the impacts of environmental variability (Falk-Petersen et al. 2009), although *C. glacialis* can revert to a single year life-cycle in certain conditions. *Calanus finmarchicus* must complete its life-cycle in a single year, and relies on open-water phytoplankton for its main source of food (Jónasdóttir et al. 2002; Melle et al. 2014). Furthermore, cold Arctic temperatures slow development rates, making it difficult to reach late developmental stages that can resource their lipid sacs sufficiently to overwinter successfully (Ji et al. 2012; Melle et al. 2014). It is considered, therefore, that *C. finmarchicus* is incapable of local recruitment within the Arctic over multiple generations (Melle et al. 2014) and its presence there is wholly dependent on being transported by northward flowing Atlantic currents (Wassmann et al. 2015).

The Fram Strait comprises a complex transition between Arctic and Atlantic water masses. Westward of this region is the dominant outflow of Arctic Ocean, comprising the East Greenland Current (EGC), which follows the east Greenland coast southwards, and a deeper outflow from the Arctic basin. To the east is an inflow of Atlantic water from the Norwegian Sea, constituting the West Spitsbergen Current (WSC), that flows northwards past the west coast of Svalbard. Warming of the Fram Strait has resulted from increased Atlantic inflow into the region (Schauer et al. 2004) which diminishes the extent of seasonal sea-ice and lengthens the productive season (Kahru et al. 2011; Polyakov et al. 2020). This in turn will alter species composition and likely affect ecosystem function.

Here, we firstly consider the historical changes that have occurred in the distribution of *C. finmarchicus* habitat within the Fram Strait region of the Arctic. Secondly, we analyse recent field data to assess the population structure and body condition of *C. finmarchicus* within these populations. Finally, we assess the potential for these animals to complete their life-cycles in this region and the implications this may have for the future of Arctic food-webs.

## Materials and methods

### Ecological niche modelling

To identify the suitability and changes in the habitat for *C. finmarchicus* in the Fram Strait, we constructed an ecological niche model (ENM) for this species, whereby occurrence and environmental data were fitted using the presence-only ENM algorithm MaxEnt v. 3.4.1 using the R package SDMtune (Vignali et al. 2020). Further details on the input data, methodology, and model performance are detailed in Electronic Supplementary Material (ESM) and in Freer et al. (2021). MaxEnt gives an estimate of the relative habitat suitability of each grid cell by comparing environmental conditions at occupied locations to the available conditions within the study region. Accordingly, suitable habitats were defined as all locations where environmental conditions match those of existing species occurrence records. Separate estimates of habitat suitability were predicted for each season; January–March, April–June, July–September, and October–December. We then compared these seasonal predictions between two ~ 30 year eras, covering the periods 1955–1984 (Era 1) and 1985–2017 (Era 2). These eras were chosen as they represent two different (cool and warm) oceanographic regimes in the northern North Atlantic known to have affected zooplankton community dynamics (Beaugrand et al. 2008). The spatial extent of the model covers the entire distribution of this species, including its northern extent in the Arctic (see ESM). For the purposes of the present study, we focus only on model projections for the Fram Strait region, and further constrain ourselves to projections for just the early productive season (April–May–June) and late productive season (July–August–September), coincident with our sampling campaigns (see below).

### Population dynamics and depth distribution

**Fig. 2:**
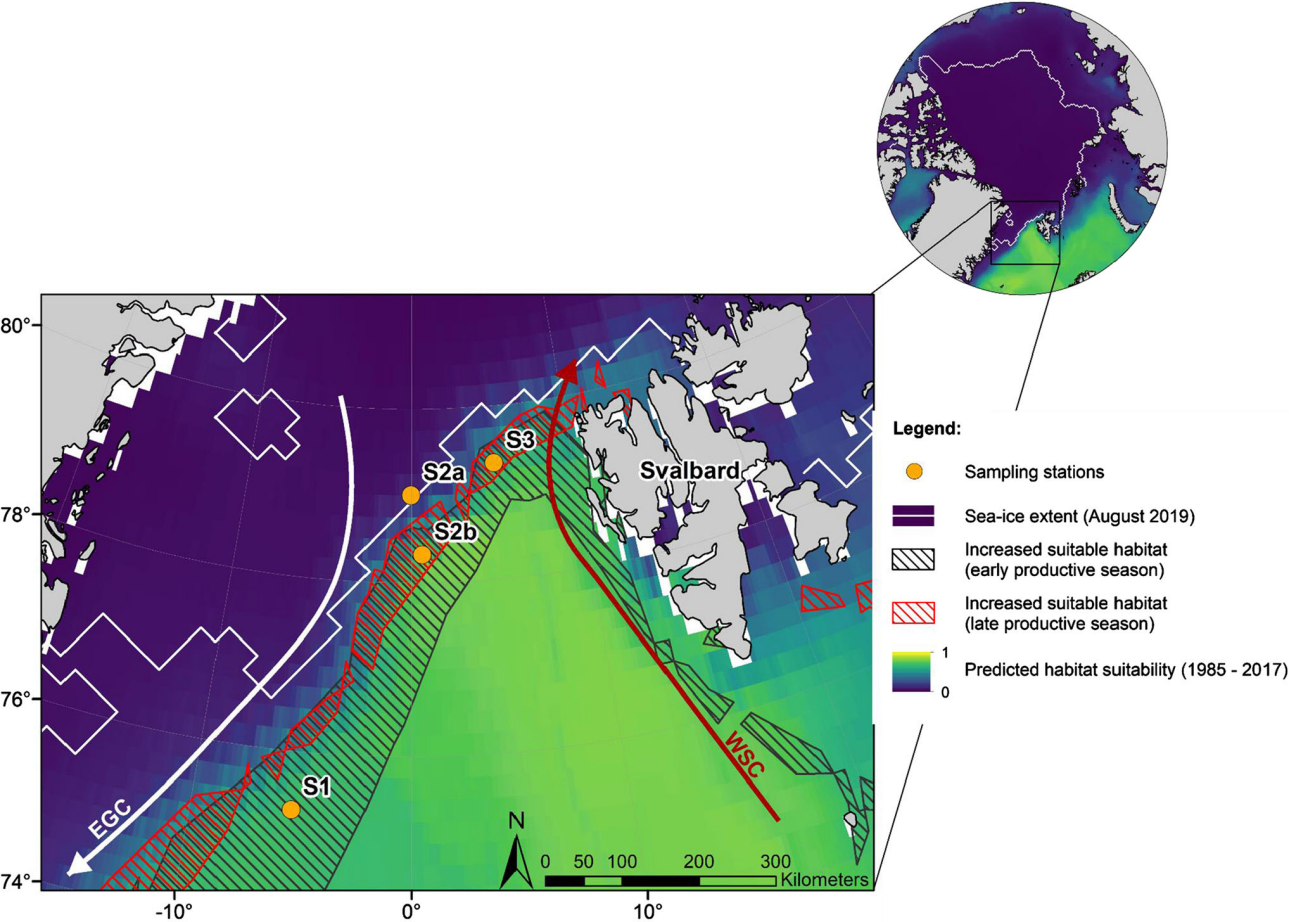
Map of the Fram Strait showing sampling stations and the projected habitat suitability for *C. finmarchicus* in the era 1985–2017, as determined by the ecological niche model we describe. Hatched areas indicate those regions where the habitat suitability has markedly increased since the previous era (1955–1984), black hatching for early productive season (April–May–June) and red hatching for late productive season (July–August–September). Sea ice extent during field sampling in August 2019 is also indicated. *WSC* West Spitsbergen Current, *EGC* East Greenland Current. Also showing location of sampling stations. Note that there were two locations for S2—S2a sampled in early summer 2018 and S2b in late summer 2019

Two multidisciplinary field campaigns were carried out in the Fram Strait region during early summer 2018 (JR17005, 08/05/2018 to 08/06/2018) and late summer 2019 (JR18007, 04/08/2019 to 28/08/2019) aboard *RRS James Clark Ross*. We analyse sampling carried out at three locations (S1, S2 and S3) spaced approximately equidistant from each other from west to east across the Fram Strait and close to the prevailing ice-edge (Fig. 2; Table 1; for S2, there were two station locations, a and b, because of prevailing ice conditions). The locations were within newly favourable habitats for *C. finmarchicus* according to the ecological niche modelling described above. At each station, a 1 m^2^ MOCNESS multinet system was deployed between the surface and a maximum depth of between 1000 and 1200 m and sampled 8 equal depth intervals of 125–150 m. All catches were preserved in buffered 10% formalin. *Calanus* species were identified to species for the CIV, CV and CVI developmental stages following size classes established by Hirche (1997). Stages CI–III were initially binned into a single “*Calanus*” category. For early summer samples, the prosome length of a random subset of 30 CI–III individuals were measured to discriminate *C. hyperboreus* from *C. glacialis/C. finmarchicus* so that the proportional representation of these two groups could be estimated.

**Table 1:** Population status of *Calanus* in the Fram Strait showing: late development stage depth integrated abundance of *C. finmarchicus*; percentage composition of three main *Calanus* species in surface waters according to 16S meta-barcoding analysis; abundance and percentage composition of *C. finmarchicus* early developmental stage (CI–III) in the surface depth interval during the early summer; and average (± SD) water column temperature in the surface 125 m. Note that S2a was sampled in early summer (2018), S2b in late summer (2019). *This was 5–1200 m in station S1 **this was 5–150 m in station S1

Station	Summer period	*C. finmarchicus* CIV, CV and CVI abundance (ind m^−2^, 5–1000 m)*		% of *C. finmarchicus* (*C.f*):*C. hyperboerus* (*C.h*):*C. glacialis* (*C.g*) from 16S meta-barcode analysis (0–200 m)		Abundance (ind m^−2^) of *C. finmarchicus* CI–III (% composition within *Calanus* CI–III community; 5–125 m**)	Average surface temperature °C ± SD (0–125 m)	
		Early	Late	Early	Late	Early	Early	Late
S1 (75.330° N, 5.466° W)		CIV: 1995	CIV: 3933	*C.f*: 63%	*C.f*: 100%	335 (33%)	0.939 ± 0.358	2.362 ± 1.589
		CV: 1283	CV: 2592	*C.h*: 36%	*C.h*: 0%			
		CVI: 703	CVI: 186	*C.g*: 1%	*C.g*: 0%			
S2a (79.003° N, 0.025° W)		CIV: 588	CIV: 6023	*C.f*: 86%	*C.f*: 100%	995 (50%)	1.017 ± 1.746	3.643 ± 1.436
S2b (78.320° N, 0.605° W)		CV: 1300	CV: 12 098	*C.h*: 11%	*C.h*: 0%			
		CVI: 812	CVI: 1765	*C.g*: 3%	*C.g*: 0%			
S3 (78.983° N, 4.366° E)		CIV: 2615	CIV: 25 061	*C.f*: 89%	*C.f*: 100%	4354 (70%)	2.750 ± 0.228	4.319 ± 1.528
		CV: 1703	CV: 15 710	*C.h*: 9%	*C.h*: 0%			
		CVI: 1827	CVI: 3989	*C.g*: 1%	*C.g*: 0%			

Further taxonomic discrimination of the three *Calanus* species was carried out with molecular analyses for which samples were taken with a 61 cm diameter Bongo net to a maximum depth of 200 m and preserved in 99% ethanol. Subsamples were analysed using a 16S ribosomal RNA gene barcode (16SAR, 16SB2R primers) (Lindeque et al. 1999) following an adapted protocol (Lindeque et al. 2013). Amplified DNA was sequenced using the Illumina high-throughput sequencing (HTS) platform. Resultant sequences processed through the Qiime pipeline, clustered into Operational Taxonomic Units at 97% homology and taxonomy was assigned using BLASTn (NCBI). For cruise JR17005, it was necessary to analyse samples from the nearest available stations to S1 and S2 which were at 75.796° N, 7.218° W and 78.998° N, 2.999° W respectively.

Full water column environmental profiles were obtained using a calibrated Sea-Bird SBE911Plus Conductivity Temperature Depth (CTD), of which analysis is provided in ESM.

### Body condition analysis

We examined the body condition of individual *C. finmarchicus* over their depth distribution through extracting approximately 10 individuals (where possible) from each MOCNESS depth-interval. Images were taken of individuals through a microscope and subsequently analysed by “image-J” software to determine various morphometric parameters including prosome length and lipid sac area (Fig. 1). Each specimen was then transferred to an individual tin capsule for subsequent elemental (carbon, hydrogen, nitrogen) analysis using a CE440 Elemental Analyser (Exeter Analytical Limited).

### Life-cycle modelling

To determine the capacity of late-summer individuals to overwinter and emerge in spring with sufficient reserves to mature and reproduce, we followed the approach of Jónasdóttir et al. (2019). Diapause duration was defined as the time it would take to respire the lipid reserve to 20% of its pre-diapause mass, following Saumweber and Durbin (2006). The model is carbon (C) based and includes estimates of structural mass, *m* which we distinguish from the lipid reserve, *w*, such that total carbon mass, *M* = *m* + *w*. It is assumed that only *m* is responsible for the active metabolism that determines respiration rate.

To calculate *m*, we firstly determined the C content of the lipid reserve. Lipid sac area derived by image analysis was converted into WEs by using the formula WE (μg) = 0.167 × *A*^1.42^ from Vogedes et al. (2010), where *A* is the area of the lipid sac in mm^2^. This value was then multiplied by 0.79 to convert to WE carbon (*w*), following Kattner and Hagen (2009). Finally, *w* was subtracted from our estimate *M*, derived from elemental analysis, to determine structural mass *m* (μg C).

Respiration rate of a diapausing *C. finmarchicus* was calculated following Visser et al. (2017):

1 $$r\left(M, T\right)=b\cdot {m}^\frac{3}{4}\mathrm{exp}\left[E\cdot \frac{\left(T-{T}_{0}\right)}{\left(k\cdot T\cdot {T}_{0}\right)}\right],$$

where *r* (μg C s^−1^) is respiration rate, *T* (K) is temperature in Kelvin, *b* (μg C^1/4^ s^−1^) is a universal scaling constant, *E* (eV) is the activation energy, and *k* (eV K^−1^) the Boltzmann constant. *T*_0_ is base temperature, taken to be *T*_0_ =  − 273 °C (absolute zero). We used universal scaling constant *b* of 2.5 × 10^−7^ μg C^1/4^ s^−1^. *r* (μg C s^−1^) was converted in units μg C day^−1^ through multiplying by seconds per day (86 400).

Diapause duration (*D*, days) for each individual included in the body condition analysis (above) was determined as follows:

2 $$D =\frac{w-(0.2\cdot w)}{r}.$$

We considered individuals capable of performing a successful diapause if *D* ≥ 150 days. This represents a conservative estimate of required diapause duration based on an average duration of 141 days (SD ± 44) across most major *C. finmarchicus* habitats using data provided in Jónasdóttir et al. (2019). We further calculated a theoretical maximum diapause duration based on the maximum possible lipid reserve as a function of prosome length, also following Jónasdóttir et al. (2019).

## Results

Our ecological niche modelling indicated a widespread increase in suitable habitat for *C. finmarchicus* since the 1980s, spanning much of the northern region of the Fram Strait stretching from the north-western tip of Svalbard and across to the Greenland Shelf (Fig. 2). Increases in suitable conditions appear to be much greater in the early compared to late productive season. This is explicable in terms of the influence of the sea-ice edge as it expands and contracts on a seasonal basis. More specifically, it is reflective of an earlier retreat of the seasonal sea-ice edge in the present era.

*Calanus* abundance increases progressively from stations S1 to S3, with highest values being observed during late summer (Table 1). 16S meta-barcoding analyses indicated that *C. glacialis* were almost a negligible part of the population in the upper 200 m at all three stations while *C. hyperboreus* were only present in this part of the water column in the early summer. *Calanus finmarchicus* made up between 63 and 89% of the *Calanus* community in the upper 200 m during the early summer and 100% across all stations in the late summer. All CI–III individuals with prosome lengths too small to be *C. hyperboreus* were assumed to be *C. finmarchicus* given the negligible levels of *C. glacialis* at these stations (Table 1). Moving from station S1 to S3, *C. finmarchicus* made up a proportionally greater part of the early summer *Calanus* CI–III population, to the point where it dominated these developmental stages at S3.

**Fig. 3:**
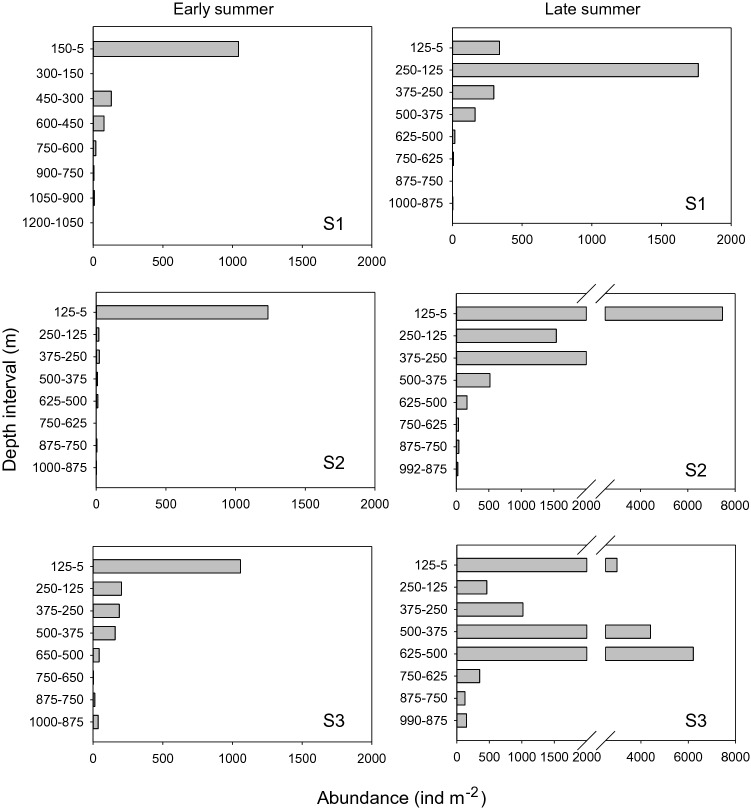
Depth distribution of *C. finmarchicus* CV during early summer (May, 2018) and late summer (August, 2019). Note that depth intervals in S1 early summer are larger by 25 m compared to other station samples

The abundance and depth distribution of *C. finmarchicus* were relatively similar across all three stations in early summer, with the large majority of the population residing between the surface and 125 to 150 m, reaching abundances of around 1000 ind m^−2^ (Fig. 3). In late summer, abundances were mainly concentrated above 250 m at S1 and S2 but, in S3, there was a second abundance peak between 625 and 375 m. Very few individuals were found below 600 m at any station in either season.

**Fig. 4:**
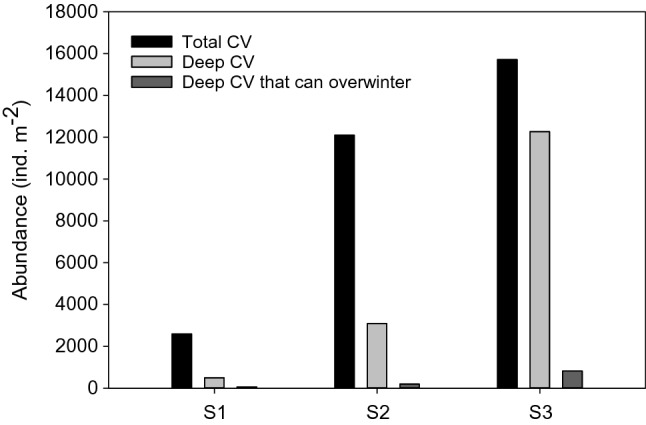
Abundance of *C. finmarchicus* CV during late summer (August, 2019) showing total abundance, deep abundance (> 250 m) and abundance of deep CV that are capable of successful overwintering

Regarding diapause lengths, we calculated that individuals in the Fram Strait needed to attain an average of 76% of their theoretical maximum lipid sac size in order to overwinter successfully and reproduce the following spring. Only 10 to 15% of deep individuals contained such lipid reserves (Table 2). However, the fact that population size at S3 was so large meant that substantive numbers (> 800 ind m^−2^) were capable of overwintering successfully (Fig. 4). This was not the case at S1 and S2, where the deep population size was much smaller.

**Table 2:** Population status of deep *Calanus finmarchicus* CV in the Fram Strait during late summer (August, 2019) showing abundance in deeper depth layer (> 250 m); average and range of the number of days over which individuals can remain in diapause; and abundance of deep CV capable of remaining in diapause for > 150 days. Calculation of diapause length was based on Jonasdottir et al. (2019) and takes account of the need to retain sufficient reserves to reproduce the following spring

	Deep CV abundance (> 250 m; ind m^−2^)	Diapause length of deep CV population (days)	Abundance of deep CV population capable of > 150 days diapause (ind m^−2^)
S1	489	Max: 184 Min: 30 Av: 112	59
S2	3095	Max: 212 Min: 41 Av: 101	192
S3	12 262	Max: 213 Min: 4 Av: 95	816

## Discussion

### Changes in the distribution of *C. finmarchicus* in the modern era

Our study indicates that, over the last 30 years, conditions within the Fram Strait have increased in suitability for *C. finmarchicus*. These areas span much of the northern region of the Fram Strait and stretch down the west coast of Svalbard and into the Barents and Greenland Seas. Our findings are in line with other studies demonstrating a northward biogeographic shift of this species’ ecological niche (Chust et al. 2014). *Calanus finmarchicus* is generally considered to have centres of distribution in the Norwegian and Labrador Seas, but it is also the dominant biomass zooplankton species south of Newfoundland, western Svalbard, the Barents Sea south of the Polar Front and the Norwegian coast (Aksnes and Blindheim 1996; Planque et al. 1997; Falk-Petersen et al. 2009). Historically, although the species has been recorded in the Arctic, numbers have been comparatively low (Hirche and Kosobokova 2007). Our recent estimates of around 15 000 ind m^−2^ CV at S3 in the late summer are nevertheless comparable with abundances in regions further south, such as the Irminger Sea, West Norwegian Sea, Labrador Sea and Iceland Sea (Heath et al. 2008; Pepin and Head 2009; Jónasdóttir et al. 2019).

Areas expected to have undergone an increase in habitat suitability for *C. finmarchicus* since the 1980s strongly overlap with regions where seasonal sea-ice in the Fram Strait has retreated over the last 30 years. In part, this is attributable to the enlarged inflow of warm water into the region (Schauer et al. 2004), bringing with it greater fluxes of salt and heat that both prevents sea-ice formation and increases ocean heat content. As a result, parts of the Fram Strait that once exhibited Arctic type cold, stratified and ice-covered features now resemble a more boreal Atlantic-type warm, well-mixed open-water system. These latter conditions are more suited to *C. finmarchicus*.

### Evidence of self-sustaining populations of *C. finmarchicus* in the Arctic

Increased habitat suitability for *C. finmarchicus* in Arctic regions such as the Fram Strait does not necessarily imply that a species recruits locally and high abundances may simply reflect a greater level of expatriation from population centres further south (Hirche and Kosobokova 2003; Daase et al. 2007; Wassmann 2011). Models have estimated that 1.5 Mt C year^−1^ of *C. finmarchicus* leave the northern Norway shelf each spring and are transported northwards, with a major flux directed to the eastern Fram Strait (Gluchowska et al. 2017). As they become advected into increasingly hostile Arctic conditions, their fate has been considered as a “trail of life and death” (Wassmann et al. 2015; Wassmann et al. 2020). This view is based on observations that the biomass of *C. finmarchicus* in the Fram Strait region decreases from October to May and does not increase again until in August/September, when a new spring cohort becomes advected into the region (Wassmann et al. 2019). The historical lack of early life-stages earlier in the year has led to the reasonable assumption that all *C. finmarchicus* in the Fram Strait and further north are allochthonous and sterile.

While *C. finmarchicus* will continue to be advected into unsuitable regions further north and persist there as sterile expatriates, in the Fram Strait we found early developmental stages (CI–III) to be a substantive part of the easternmost station (S3) in early summer. Weydmann et al. (2018) considered the composition of *C. finmarchicus* life-stages in the WSC to the west of Svalbard and found early stages to be present in mid-summer (late June, early July). Their consideration was that females that originated from the Norwegian shelf spring cohort spawned them. However, early stages occurring in May and early June must otherwise be spawned from a local overwintering population since it is substantially in advance of the mid to late summer influx.

Spawning in the Fram Strait from a local overwintering population is further supported by our observation that 10 and 15% of the deep population at S3 were capable of completing the final part of their life cycle of surviving the winter and reproducing the following spring. *Calanus finmarchicus* have a high fecundity with egg production rates often between 10 and 50 eggs per day (Melle et al. 2014). Even if these viable individuals comprise just a comparatively small part of the Fram Strait population, optimal timing of their reproductive efforts with the spring bloom could enable them to support a substantial resident population in this region (Varpe et al. 2007).

The abundance of early season CI–III is equivalent to just 10% of the of the total population size of *C. finmarchicus* found at this location in late summer, indicating that inward advection of *C. finmarchicus* remains the major contributor to the late development stage population found in this region. Nevertheless, our discovery of viable overwintering individuals and locally recruited early developmental stages together provide the first empirical evidence that *C. finmarchicus* is now capable of completing its life-cycle at ice-edge locations in the Fram Strait.

**Fig. 5:**
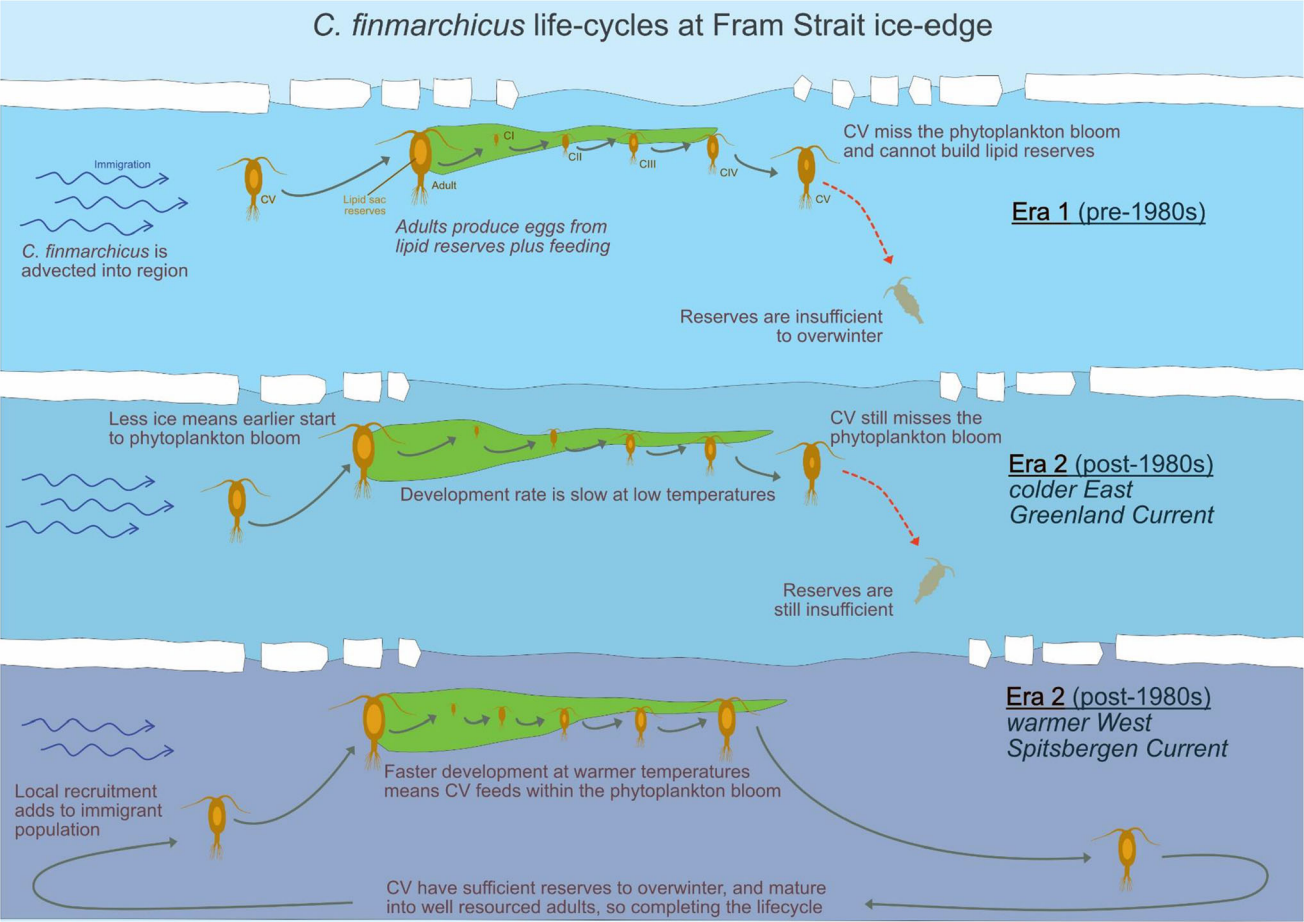
Schematic life-cycle of *C. finmarchicus* occurring in the Fram Strait in the 30 year era leading up to the mid 1980s (Era 1) and the subsequent 30 years (Era 2). In Era 1, the *C. finmarchicus* population entirely relied on immigration from regions further south in order to populate the region. The short period of open water after the break-up of ice means the phytoplankton bloom is comparatively late and brief. *C. finmarchicus* are unable to develop sufficiently quickly in the cold temperatures and build up sufficient reserves to overwinter. In Era 2, increased inflow of warm Atlantic water means less ice and a longer productivity season. Nevertheless, in those regions most influenced by the cold East Greenland Current, slow development means that the late developmental stages miss ideal feeding conditions and sufficient overwintering reserves cannot be attained. In Era 2, where the warm Atlantic inflow of the West Spitsbergen Current has greater influence, development is more rapid and late developmental stages feed within the bloom, facilitating the build-up sufficient overwintering reserves. This enables a new cohort to be spawned at the start of the phytoplankton bloom the following spring. Nevertheless, immigration of late stage *C. finmarchicus* from further south still remains an important input into the Fram Strait population

While all our stations were in an area from which seasonal sea-ice has retreated since the 1980s, strong evidence that *C. finmarchicus* could complete its life-cycle was found only at the most easterly station (S3) where surface temperatures were around 2 °C warmer than at the westerly station (S1). These warmer temperatures likely increase development rates through the earlier stages of the life-cycle meaning late overwintering developmental stages will also be reached sooner, giving them greater opportunity to feed and build up lipid reserves prior to overwintering. Furthermore, abundance and nutritional quality of phytoplankton is higher in the earlier part of the productive season (Leu et al. 2011), giving further advantage to these faster developing individuals. Thus, both decreasing sea ice extent and increasing water temperature provide favourable conditions that will enable *C. finmarchicus* to complete their life-cycle (Fig. 5) and since these environmental conditions are becoming more prevalent (Polyakov et al. 2017), *C. finmarchicus* is likely to establish itself further in this region.

Pelagic populations exist within an advective environment where the influx and outflow of individuals to and from other ocean regions occurs continuously and individuals often do not remain within a single region over their entire lifespans. In this context, our study shows that sufficient *C. finmarchicus* adults now complete their life-cycle while moving through the Fram Strait to produce a distinguishable population of offspring. As conditions become even more favourable, life-cycle completion will be achieved by more adults, producing more offspring and making this region into a source rather than a sink for this species.

### Implications to Arctic ecosystems

Our findings that *C. finmarchicus* now complete their life-cycle within the Fram Strait may indicate a shift towards this small boreal species taking over dominance from the larger Arctic species (*C. hyperboreus*, *C. glacialis*). Such a shift to a smaller species, with a correspondingly smaller lipid sac, may have the effect of decreasing the size of the available lipid pool for planktivores and higher predators. Models have indicated that the resulting faster generation times and population turnover rates of this smaller species may compensate with regards to energy transfer to predators (Renaud et al. 2018). Nevertheless, the change in prey size may impact predators that target individual *Calanus*, such as little auks (*Alle alle*) who actively select larger *Calanus* (Karnovsky et al. 2003; Kwasniewski et al. 2012; Vogedes et al. 2014).

A *Calanus* species shift may bring further changes in terms of the timing of respective life-cycles. The timing of diapause and reproduction may differ between *Calanus* species in the Arctic by one to two months (Madsen et al. 2001). This, in turn, alters the seasonal availability of a particular *Calanus* life-stage to key predators, affecting the ability of these predators to remain resident in these regions. For instance, the biomass of polar cod in the Barents Sea, which neighbours the Fram Strait, has declined dramatically since 2006 (Hop and Gjøsæter 2013). The young stages of that fish species have a strong preference for eggs and larvae of *C. glacialis* (Bouchard and Fortier 2020), which are available prior to the spring bloom and in close association with the ice (Daase et al. 2013). By contrast, boreal fish species, such as capelin, Atlantic cod and mackerel are increasing in the Barents Sea and further north (Berge et al. 2015; Fossheim et al. 2015; Kortsch et al. 2015). The young of those species thrive on eggs and larvae of *C. finmarchicus* (Bjørke 1976; Heath and Lough 2007) which are more closely synchronised to the open-water spring bloom (Melle et al. 2014). Fish stocks that are more capable of matching the timing of their young stages with that of its principal food are the ones most likely to continue to succeed as these systems change (Cushing 1990). With greater levels of open water in the Fram Strait, this temporal matching may well increasingly favour boreal fish species.

Polar ecosystems such as the Fram Strait can be considered as sentinel systems for the wider fate of polar regions. As the region experiences greater encroachment of warm Atlantic currents and the retreat of seasonal sea-ice, what was a minor expatriate population of *C. finmarchicus* in decades past has now become reproductively viable of this region. This has implications not only for other Arctic species in this region, but also to species such as large baleen whales (blue, fin, bowhead, humpback) that migrate in and out of this region to exploit its rich feeding grounds (Moore et al. 2019). A *Calanus* species shift is also likely to facilitate further northward expansion of commercially exploited fish stocks. We must manage our further interactions with this system carefully to avoid exceeding the tolerance of an ecosystem already undergoing rapid change.

## Supplementary Information

Below is the link to the electronic supplementary material.

Supplementary file1 (PDF 649 kb)

## References

[CR1] Aarflot JM, Skjoldal HR, Dalpadado P, Skern-Mauritzen M (2018). Contribution of *Calanus* species to the mesozooplankton biomass in the Barents Sea. ICES Journal of Marine Science.

[CR2] Aksnes D, Blindheim J (1996). Circulation patterns in the North Atlantic and possible impact on population dynamics of *Calanus finmarchicus*. Ophelia.

[CR3] Årthun M, Eldevik T, Smedsrud L, Skagseth Ø, Ingvaldsen R (2012). Quantifying the influence of Atlantic heat on Barents Sea ice variability and retreat. Journal of Climate.

[CR4] Beaugrand G, Edwards M, Brander K, Luczak C, Ibanez F (2008). Causes and projections of abrupt climate-driven ecosystem shifts in the North Atlantic. Ecology Letters.

[CR5] Berge J, Heggland K, Lønne OJ, Cottier F, Hop H, Gabrielsen GW, Nøttestad L, Misund OA (2015). First records of Atlantic mackerel (*Scomber scombrus*) from the Svalbard Archipelago, Norway, with possible explanations for the extensions of its distribution. Arctic.

[CR6] Bjørke, H. 1976. *Some preliminary results on food and feeding of young capelin larvae*. ICES 1976/H:37.

[CR7] Bouchard C, Fortier L (2020). The importance of *Calanus glacialis* for the feeding success of young polar cod: A circumpolar synthesis. Polar Biology.

[CR8] Chust G, Castellani C, Licandro P, Ibaibarriaga L, Sagarminaga Y, Irigoien X (2014). Are *Calanus* spp. shifting poleward in the North Atlantic? A habitat modelling approach. ICES Journal of Marine Science.

[CR9] Cushing, D. 1990. Plankton production and year-class strength in fish populations: An update of the match/mismatch hypothesis. In: *Advances in marine biology*, vol 26, 249–293. New York: Elsevier.

[CR11] Daase M, Vik JO, Bagøien E, Stenseth NC, Eiane K (2007). The influence of advection on *Calanus* near Svalbard: Statistical relations between salinity, temperature and copepod abundance. Journal of Plankton Research.

[CR10] Daase M, Falk-Petersen S, Varpe Ø, Darnis G, Søreide JE, Wold A, Leu E, Berge J (2013). Timing of reproductive events in the marine copepod *Calanus glacialis*: A pan-Arctic perspective. Canadian Journal of Fisheries and Aquatic Sciences.

[CR12] Ershova E, Kosobokova K, Banas N, Ellingsen I, Niehoff B, Hildebrandt N, Hirche HJ (2021). Sea ice decline drives biogeographical shifts of key *Calanus* species in the central Arctic Ocean. Global Change Biology.

[CR13] Falk-Petersen S, Mayzaud P, Kattner G, Sargent J (2009). Lipids and life strategy of Arctic *Calanus*. Marine Biology Research.

[CR14] Fossheim M, Primicerio R, Johannesen E, Ingvaldsen RB, Aschan MM, Dolgov AV (2015). Recent warming leads to a rapid borealization of fish communities in the Arctic. Nature Climate Change.

[CR15] Freer J, Daase M, Tarling GA (2021). Modelling the biogeographic boundary shift of *Calanus finmarchicus* reveals drivers of Arctic ‘Atlantification’ by sub-Arctic zooplankton. Global Change Biology.

[CR16] Gluchowska M, Dalpadado P, Beszczynska-Möller A, Olszewska A, Ingvaldsen RB, Kwasniewski S (2017). Interannual zooplankton variability in the main pathways of the Atlantic water flow into the Arctic Ocean (Fram Strait and Barents Sea branches). ICES Journal of Marine Science.

[CR17] Heath M, Lough R (2007). A synthesis of large-scale patterns in the planktonic prey of larval and juvenile cod (*Gadus morhua*). Fisheries Oceanography.

[CR18] Heath M, Rasmussen J, Ahmed Y, Allen J, Anderson CIH, Brierley AS, Brown L, Bunker A (2008). Spatial demography of *Calanus finmarchicus* in the Irminger Sea. Progress in Oceanography.

[CR22] Hirche HJ (1996). Diapause in the marine copepod, *Calanus finmarchicus*—A review. Ophelia.

[CR19] Hirche H-J (1997). Life cycle of the copepod *Calanus hyperboreus* in the Greenland Sea. Marine Biology.

[CR20] Hirche H-J, Kosobokova K (2003). Early reproduction and development of dominant calanoid copepods in the sea ice zone of the Barents Sea—Need for a change of paradigms?. Marine Biology.

[CR21] Hirche H-J, Kosobokova K (2007). Distribution of *Calanus finmarchicus* in the northern North Atlantic and Arctic Ocean—Expatriation and potential colonization. Deep Sea Research Part II: Topical Studies in Oceanography.

[CR23] Hop H, Gjøsæter H (2013). Polar cod (*Boreogadus saida*) and capelin (*Mallotus villosus*) as key species in marine food webs of the Arctic and the Barents Sea. Marine Biology Research.

[CR24] Ji R, Ashjian CJ, Campbell RG, Chen C, Gao G, Davis CS, Cowles GW, Beardsley RC (2012). Life history and biogeography of *Calanus* copepods in the Arctic Ocean: An individual-based modeling study. Progress in Oceanography.

[CR25] Jónasdóttir S, Gudfinnsson H, Gislason A, Astthorsson O (2002). Diet composition and quality for *Calanus finmarchicus* egg production and hatching success off south-west Iceland. Marine Biology.

[CR26] Jónasdóttir SH, Wilson RJ, Gislason A, Heath MR (2019). Lipid content in overwintering *Calanus finmarchicus* across the subpolar eastern North Atlantic Ocean. Limnology and Oceanography.

[CR27] Kahru M, Brotas V, Manzano-Sarabia M, Mitchell B (2011). Are phytoplankton blooms occurring earlier in the Arctic?. Global Change Biology.

[CR28] Karnovsky NJ, Kwaśniewski S, Węsławski JM, Walkusz W, Beszczyńska-Möller A (2003). Foraging behavior of little auks in a heterogeneous environment. Marine Ecology Progress Series.

[CR29] Kattner, G., & W. Hagen. 2009. Lipids in marine copepods: Latitudinal characteristics and perspective to global warming. In: *Lipids in aquatic ecosystems*, 257–280. Berlin: Springer.

[CR30] Kortsch S, Primicerio R, Fossheim M, Dolgov AV, Aschan M (2015). Climate change alters the structure of Arctic marine food webs due to poleward shifts of boreal generalists. Proceedings of the Royal Society B: Biological Sciences.

[CR31] Kwasniewski S, Gluchowska M, Walkusz W, Karnovsky NJ, Jakubas D, Wojczulanis-Jakubas K, Harding AMA, Goszczko I (2012). Interannual changes in zooplankton on the West Spitsbergen Shelf in relation to hydrography and their consequences for the diet of planktivorous seabirds. ICES Journal of Marine Science.

[CR32] Leu E, Søreide J, Hessen D, Falk-Petersen S, Berge J (2011). Consequences of changing sea-ice cover for primary and secondary producers in the European Arctic shelf seas: Timing, quantity, and quality. Progress in Oceanography.

[CR33] Lind S, Ingvaldsen RB, Furevik T (2018). Arctic warming hotspot in the northern Barents Sea linked to declining sea-ice import. Nature Climate Change.

[CR34] Lindeque PK, Harris RP, Jones MB, Smerdon GR (1999). Simple molecular method to distinguish the identity of *Calanus* species (Copepoda: Calanoida) at any developmental stage. Marine Biology.

[CR35] Lindeque PK, Parry HE, Harmer RA, Somerfield PJ, Atkinson A (2013). Next generation sequencing reveals the hidden diversity of zooplankton assemblages. PLoS ONE.

[CR36] Madsen S, Nielsen T, Hansen B (2001). Annual population development and production by *Calanus finmarchicus*, *C. glacialis* and *C. hyperboreus* in Disko Bay, western Greenland. Marine Biology.

[CR37] Melle W, Runge J, Head E, Plourde S, Castellani C, Licandro P, Pierson J, Jonasdottir S (2014). The North Atlantic Ocean as habitat for *Calanus finmarchicus*: Environmental factors and life history traits. Progress in Oceanography.

[CR38] Miller CB, Morgan CA, Prahl FG, Sparrow MA (1998). Storage lipids of the copepod *Calanus finmarchicus* from Georges Bank and the Gulf of Maine. Limnology and Oceanography.

[CR39] Møller EF, Nielsen TG (2020). Borealization of Arctic zooplankton—Smaller and less fat zooplankton species in Disko Bay, Western Greenland. Limnology and Oceanography.

[CR40] Moore SE, Haug T, Víkingsson GA, Stenson GB (2019). Baleen whale ecology in Arctic and sub-Arctic seas in an era of rapid habitat alteration. Progress in Oceanography.

[CR41] Pepin P, Head EJH (2009). Seasonal and depth-dependent variations in the size and lipid contents of stage 5 copepodites of *Calanus finmarchicus* in the waters of the Newfoundland Shelf and the Labrador Sea. Deep-Sea Research Part I-Oceanographic Research Papers.

[CR42] Planque B, Hays GC, Ibanez F, Gamble JC (1997). Large scale spatial variations in the seasonal abundance of *Calanus finmarchicus*. Deep-Sea Research Part I-Oceanographic Research Papers.

[CR44] Polyakov IV, Pnyushkov AV, Alkire MB, Ashik IM, Baumann TM, Carmack EC, Goszczko I, Guthrie J (2017). Greater role for Atlantic inflows on sea-ice loss in the Eurasian Basin of the Arctic Ocean. Science.

[CR43] Polyakov IV, Alkire MB, Bluhm BA, Brown KA, Carmack EC, Chierici M, Danielson SL, Ellingsen I (2020). Borealization of the Arctic Ocean in response to anomalous advection from sub-Arctic seas. Frontiers in Marine Science.

[CR45] Renaud PE, Daase M, Banas NS, Gabrielsen TM, Søreide JE, Varpe Ø, Cottier F, Falk-Petersen S (2018). Pelagic food-webs in a changing Arctic: A trait-based perspective suggests a mode of resilience. ICES Journal of Marine Science.

[CR46] Saumweber WJ, Durbin EG (2006). Estimating potential diapause duration in *Calanus finmarchicus*. Deep-Sea Research Part II-Topical Studies in Oceanography.

[CR47] Schauer U, Fahrbach E, Osterhus S, Rohardt G (2004). Arctic warming through the Fram Strait: Oceanic heat transport from 3 years of measurements. Journal of Geophysical Research: Oceans.

[CR48] Søreide JE, Falk-Petersen S, Hegseth EN, Hop H, Carroll ML, Hobson KA, Blachowiak-Samolyk K (2008). Seasonal feeding strategies of *Calanus* in the high-Arctic Svalbard region. Deep Sea Research Part II: Topical Studies in Oceanography.

[CR49] Stroeve J, Notz D (2018). Changing state of Arctic sea ice across all seasons. Environmental Research Letters.

[CR50] Varpe O, Jorgensen C, Tarling GA, Fiksen O (2007). Early is better: Seasonal egg fitness and timing of reproduction in a zooplankton life-history model. Oikos.

[CR51] Vignali S, Barras AG, Arlettaz R, Braunisch V (2020). SDMtune: An R package to tune and evaluate species distribution models. Ecology and Evolution.

[CR52] Visser AW, Grønning J, Jónasdóttir SH (2017). *Calanus hyperboreus* and the lipid pump. Limnology and Oceanography.

[CR54] Vogedes D, Varpe Ø, Søreide JE, Graeve M, Berge J, Falk-Petersen S (2010). Lipid sac area as a proxy for individual lipid content of Arctic calanoid copepods. Journal of Plankton Research.

[CR53] Vogedes D, Eiane K, Båtnes AS, Berge J (2014). Variability in *Calanus* spp. abundance on fine-to mesoscales in an Arctic Fjord: Implications for little auk feeding. Marine Biology Research.

[CR55] Wassmann P (2011). Arctic marine ecosystems in an era of rapid climate change. Progress in Oceanography.

[CR57] Wassmann P, Kosobokova KN, Slagstad D, Drinkwater KF, Hopcroft RR, Moore SE, Ellingsen I, Nelson RJ (2015). The contiguous domains of Arctic Ocean advection: Trails of life and death. Progress in Oceanography.

[CR58] Wassmann P, Slagstad D, Ellingsen I (2019). Advection of mesozooplankton into the northern Svalbard shelf region. Frontiers in Marine Science.

[CR56] Wassmann P, Carmack EC, Bluhm BA, Duarte CM, Berge J, Brown K, Grebmeier JM, Holding J (2020). Towards a unifying pan-Arctic perspective: A conceptual modelling toolkit. Progress in Oceanography.

[CR59] Weydmann A, Carstensen J, Goszczko I, Dmoch K, Olszewska A, Kwasniewski S (2014). Shift towards the dominance of boreal species in the Arctic: Inter-annual and spatial zooplankton variability in the West Spitsbergen Current. Marine Ecology Progress Series.

[CR60] Weydmann A, Walczowski W, Carstensen J, Kwaśniewski S (2018). Warming of Subarctic waters accelerates development of a key marine zooplankton *Calanus finmarchicus*. Global Change Biology.

