# Supplementary material for: Can a key boreal Calanus copepod species now complete its life-cycle in the Arctic? Evidence and implications for Arctic food-webs
Source: Ambio. 2021 Nov 29;51(2):333–44. doi: 10.1007/s13280-021-01667-y (PMC8692626; doi:10.1007/s13280-021-01667-y)
Supplement: Supplementary file 1 — Supplementary file1 (PDF 649 kb) [file 13280_2021_1667_MOESM1_ESM.pdf]

Electronic Supplementary Material
This supplementary material has not been peer reviewed.

Title: **Has a key boreal *Calanus* copepod species become an Arctic resident? Evidence and implications for Arctic food-webs**

1. Analysis of overlying water masses

Full water column environmental profiles were obtained using a calibrated Sea-Bird SBE911Plus Conductivity Temperature Depth (CTD) at three stations across the Fram Strait: S1 (75.330°N, 5.466°W); S2 (79.003°N, 0.025°W); S3 (78.983°N, 4.366°E). The stations were sampled in two separate cruise, one in May-June 2018 and the other in August 2019. Here we consider the vertical water mass distribution for each pair of stations (2018/2019) working east from S1 to S3. We use the water mass descriptions of Rudels et al (2005), but make no distinction between the numerous deep water types that can be identified. At S1 (Figure S1a) in 2018, there is no clearly developed surface layer with a very weak density gradient. The water column is dominated by waters of Arctic Intermediate Water overlaying Nordic Sea Deep Water. The situation in 2019 is similar other than a warm surface layer with strong halo and themoclines to the Arctic Intermediate Water. At S2 (Figure S1b) in 2018 a cold, polar surface layer was observed, likely due to sea ice melt. This transitioned to a clear Atlantic Water layer overying Arctic Intermediate Waters with an Arctic Deep Water mass at the bottom. In 2019 the surface layer is very warm with a higher salinity than 2018, possibly a differing sea ice condition between those years. At S3 (Figure S1c) in 2018 the Atlantic Water layer is relatively close to the surface with little evidence of a sea ice melt layer. This transitions to Intermediate Waters and an Arctic Deep Water. In 2019 there is a very relatively warm and saline surface layer and evidence of interleaving effects in the Atlantic Water layer, possibly interactions with coastal waters.

Reference:

Rudels, B., Björk, G., Nilsson, J., Winsor, P., Lake, I., & Nohr, C. (2005). The interaction between waters from the Arctic Ocean and the Nordic Seas north of Fram Strait and along the East Greenland Current: results from the Arctic Ocean-02 Oden expedition. Journal of Marine Systems, 55(1-2), 1-30.

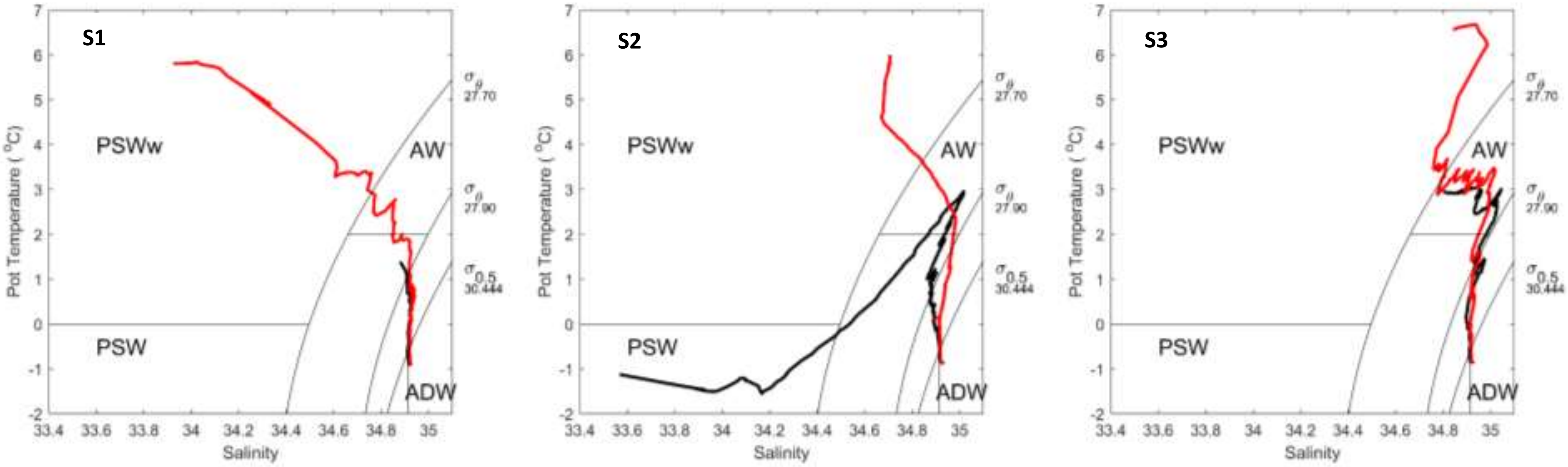

Figure S1: Temperature-Salinity curves for pairs of stations sampled in 2018 (red) and 2019 (black) within the Nordic seas at a) S1, b) S2 and c) S3. Water mass boundaries are as described in Rudels et al (2005) with Atlantic Water (AW), Arctic Deep Waters (ArDW), Polar Surface Water (PSW and warm Polar Surface Water (PSWw) noted.

2. Ecological niche modelling methods

2.1. Occurrence data

Six online repositories (OBIS, PANGAEA, NSF Arctic Data Center, BODC, COPEPOD global plankton database, NOAA NODC), comprising of 46 individual datasets, were used to compile 65,037 georeferenced occurrence records of *Calanus finmarchicus* lifestages CI-adult. Duplicate records were removed, and the remaining records were spatially thinned to remove the fewest records necessary to substantially reduce the effects of sampling bias, while simultaneously retaining the greatest amount of useful information (Aiello-Lammens et al. 2015). Thinning distance was equal to environmental data resolution (0.25° x 0.25°) and the final number of records used in analyses was 27,726 (Figure S2a).

2.2. Environmental data

Ten sea-surface environmental predictors were identified as candidate variables for the niche model (Table S1). For each predictor, seasonal climatologies from 30° - 90°N were obtained for two eras of approximately 30 years; 1955-1984 and 1985-2017. Based on data availability, the seasonal partitions represent the months; Jan-Feb-Mar (JFM), Apr-May-Jun (AMJ), Jul-Aug-Sep (JAS), and Oct-Nov-Dec (OND). Occurrence data were then matched to environmental predictor data from the corresponding season and time period in which they were collected from by adapting the method and code from (Duffy & Chown 2017).

2.3. MaxEnt niche model

Data were fitted to the presence-only ecological niche modelling algorithm MaxEnt v. 3.4.1 (Phillips & Dudik 2008, Phillips et al. 2017) using the SDMtune R package (Vignali et al. 2020). MaxEnt estimates the conditional probability of presence of a species relative to locations where the species has been observed by sampling the environment at a range of "background" locations across the study region and discriminating these from environments at locations the species is known to occupy.

A kernel density surface of zooplankton data was used to weight the selection of 10,000 random points across the region (i.e. more points taken from areas with higher density values; Figure S2b). Zooplankton data were taken as all OBIS-stored occurrence records of the phylum "arthropoda" from the upper 200m collected between 1960 and 2017 in the region of interest. These points were randomly assigned to a season and time period which was proportionate to the temporal distribution of zooplankton data. As with occurrence records, background points were subsequently matched to corresponding environmental data.

The spatialBlock function of the blockCV package in R (Valavi et al. 2019) was used to create fold partitions (k = 5) that account for spatial autocorrelation in the environmental data. Spatial block size was determined by fitting isotropic variogram models using 5000 random points from each environmental predictor raster. This finds the effective range of spatial autocorrelation and the spatial block size was based on median of these ranges. The occurrence and background data within each block were then assigned to a fold. The MaxEnt model is then run several times, withholding a different fold for evaluation each time.

To prevent model overfitting, several tuning procedures were followed and the Area Under the Receiving operator Curve (AUC) and True skill Statistic (TSS) metrics were used to evaluate model discriminatory performance on the evaluation (test) fold. First, the gridsearch function of SDMtune package was used to find optimal combination of MaxEnt hyperparameters. Models were run with varying combinations of regularisation parameter

(0.2 - 3) and iteration number parameter (300 - 900). Feature class settings did not vary with only linear and quadratic transformations allowed. Average AUC_{TEST} and TSS_{TEST} metrics were stored for each parameter combination and the one with highest values was considered optimal. Secondly, the varSel function was used to remove any correlated environmental predictors, choosing to remove whichever reduced model permutation importance the least. Thirdly, the reduceVar function was used to find and remove environmental predictors with low model contribution (<3% permutation importance).

2.4. Predicting habitat change

The optimised MaxEnt model was used to predict the habitat suitability of *C. finmarchicus* across the region of interest. Separate predictions were made for each season (JFM, AMJ, JAS, OND) and era (1955-1984, 1985-2017). To assess changes in the spatial pattern of habitat suitability between the two eras, we subtracted model predictions of habitat suitability for the most recent era from the former and repeated this calculation for each season. Here, we focus on the change in habitat suitability for the early productive season (AMJ) and late productive season (JAS) clipped to the Fram Strait region.

2.5. Results summary

The final model, optimised in terms of MaxEnt hyperparameters and environmental predictors, retained five predictors (in order of permutation importance: sea-ice concentration, temperature, salinity, chlorophyll a, bathymetry) and had good discriminatory ability (mean AUC_{TEST} = 0.72, mean TSS_{TEST} = 0.41). The model predicts that *C. finmarchicus* habitat is characterized by optimal surface temperatures between 4 and 12°C, with a peak at 9°C. This corresponds to a geographic distribution spanning 40-80°N, with high suitability across the North Atlantic and Norwegian Sea, and southern and northern range edges that vary seasonally (Fig. S3a-b). This biogeography is consistent with previous model findings (Helaouet & Beaugrand 2007, Helaouet & Beaugrand 2009, Beaugrand et al. 2013, Albouy-Boyer et al. 2016) and observations (Bonnet et al. 2005, Strand et al. 2020). Moreover, our results demonstrate that suitable habitat for *C. finmarchicus* has increased at Arctic latitudes in the last 30 years while temperate regions have declined in suitability (Fig S3c-d). This is in line with observations of boreal species entering, and becoming more dominant within, Arctic ecosystems (Kortsch et al. 2012, Fossheim et al. 2015, Aarflot et al. 2018, Dalpadado et al. 2020, Moller & Nielsen 2020, Polyakov et al. 2020). Full assessment of model results are within Freer et al. (In press).

References:

Aarflot JM, Skjoldal HR, Dalpadado P, Skern-Mauritzen M (2018) Contribution of Calanus species to the mesozooplankton biomass in the Barents Sea. ICES J Mar Sci 75:2342-2354

Aiello-Lammens, M.E., Boria, R.A., Radosavljevic, A., Vilela, B., Anderson, R.P. (2015). spThin: an R package for spatial thinning of species occurrence records for use in ecological niche models. Ecography 38: 541-545.

Albouy-Boyer S, Plourde S, Pepin P, Johnson CL, Lehoux C, Galbraith PS, Hebert D, Lazin G, Lafleur C (2016) Habitat modelling of key copepod species in the Northwest Atlantic Ocean based on the Atlantic Zone Monitoring Program. J Plankton Res 38:589-603

Beaugrand G, Mackas D, Goberville E (2013) Applying the concept of the ecological niche and a macroecological approach to understand how climate influences zooplankton: Advantages, assumptions, limitations and requirements. Prog Oceanogr 111:75-90

Bonnet D, Richardson A, Harris R, Hirst A, Beaugrand G, Edwards M, Ceballos S, Diekman R, Lopez-Urrutia A, Valdes L, Carlotti F, Molinero JC, Weikert H, Greve W, Lucic D, Albaina A, Yahia ND, Umani SF, Miranda A, dos Santos A, Cook K, Robinson S, de Puelles MLF (2005) An overview of Calanus helgolandicus ecology in European waters. Prog Oceanogr 65:1-53

Dalpadado P, Arrigo KR, van Dijken GL, Skjoldal HR, Bagoien E, Dolgov AV, Prokopchuk IP, Sperfeld E (2020) Climate effects on temporal and spatial dynamics of phytoplankton and zooplankton in the Barents Sea. Prog Oceanogr 185

Duffy, G.A., Chown, S.L. (2017). Explicitly integrating a third dimension in marine species distribution modelling. Marine Ecology Progress Series, 564: 1-8.

Fossheim M, Primicerio R, Johannesen E, Ingvaldsen RB, Aschan MM, Dolgov AV (2015) Recent warming leads to a rapid borealization of fish communities in the Arctic. Nature Climate Change 5:673-677

Freer, J. J. et al. Modelling the biogeographic boundary shift of *Calanus finmarchicus* reveals drivers of Arctic 'Atlantification' by subarctic zooplankton (In press).

Helaouet P, Beaugrand G (2007) Macroecology of Calanus finmarchicus and C-helgolandicus in the North Atlantic Ocean and adjacent seas. Mar Ecol Prog Ser 345:147-165

Helaouet P, Beaugrand G (2009) Physiology, Ecological Niches and Species Distribution. Ecosystems 12:1235-1245

Kortsch S, Primicerio R, Beuchel F, Renaud PE, Rodrigues J, Lonne OJ, Gulliksen B (2012) Climate-driven regime shifts in Arctic marine benthos. Proceedings of the National Academy of Sciences of the United States of America 109:14052-14057

Moller EF, Nielsen TG (2020) Borealization of Arctic zooplankton-smaller and less fat zooplankton species in Disko Bay, Western Greenland. Limnol Oceanogr 65:1175-1188

Phillips, S.J., Anderson, R.P., Dudik, M., Schapire, R.E., Blair, M.E. (2017). Opening the black box: an open-source release of Maxent. Ecography 40: 887-893.

Phillips SJ, Dudik M (2008) Modeling of species distributions with Maxent: new extensions and a comprehensive evaluation. Ecography 31:161-175

Polyakov IV, Alkire MB, Bluhm BA, Brown KA, Carmack EC, Chierici M, Danielson SL, Ellingsen I, Ershova EA, Gardfeldt K, Ingvaldsen RB, Pnyushkov AV, Slagstad D,

Strand E, Bagoien E, Edwards M, Broms C, Klevjer T (2020) Spatial distributions and seasonality of four Calanus species in the Northeast Atlantic. Prog Oceanogr 185

Valavi, R., Elith, J., Lahoz-Monfort, J.J., Guillera-Arroita, G. (2019). blockCV: An r package for generating spatially or environmentally separated folds for k-fold cross-validation of species distribution models. Methods in Ecology and Evolution, 10: 225-232.

Vignali, S., Barras, A.G., Arlettaz, R., Braunisch, V. (2020). SDMtune: An R package to tune and evaluate species distribution models. Ecology and Evolution, 10: 11488-11506.

Wassmann P (2020) Borealization of the Arctic Ocean in Response to Anomalous Advection From Sub-Arctic Seas. Frontiers in Marine Science 7

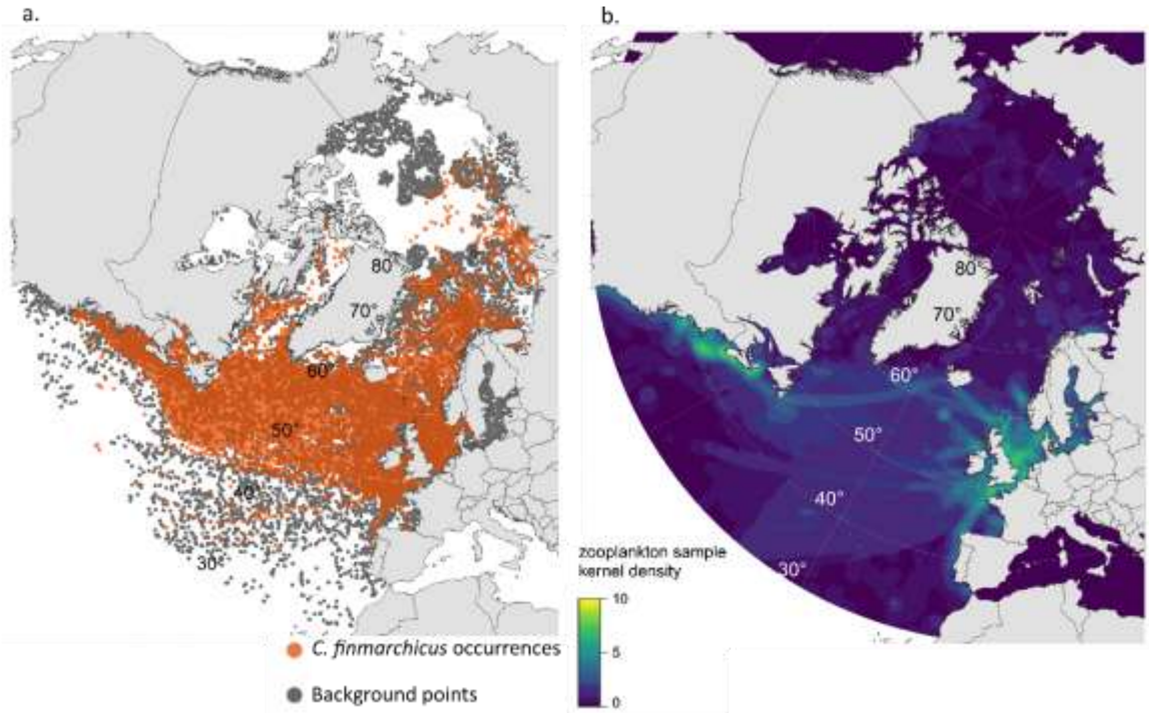

Figure S2: The presence and background points required for MaxEnt modelling (a.) and a kernel density map of sampling effort based on accessible records of zooplankton in the upper 200m (b.) which was used to weight background point selection.

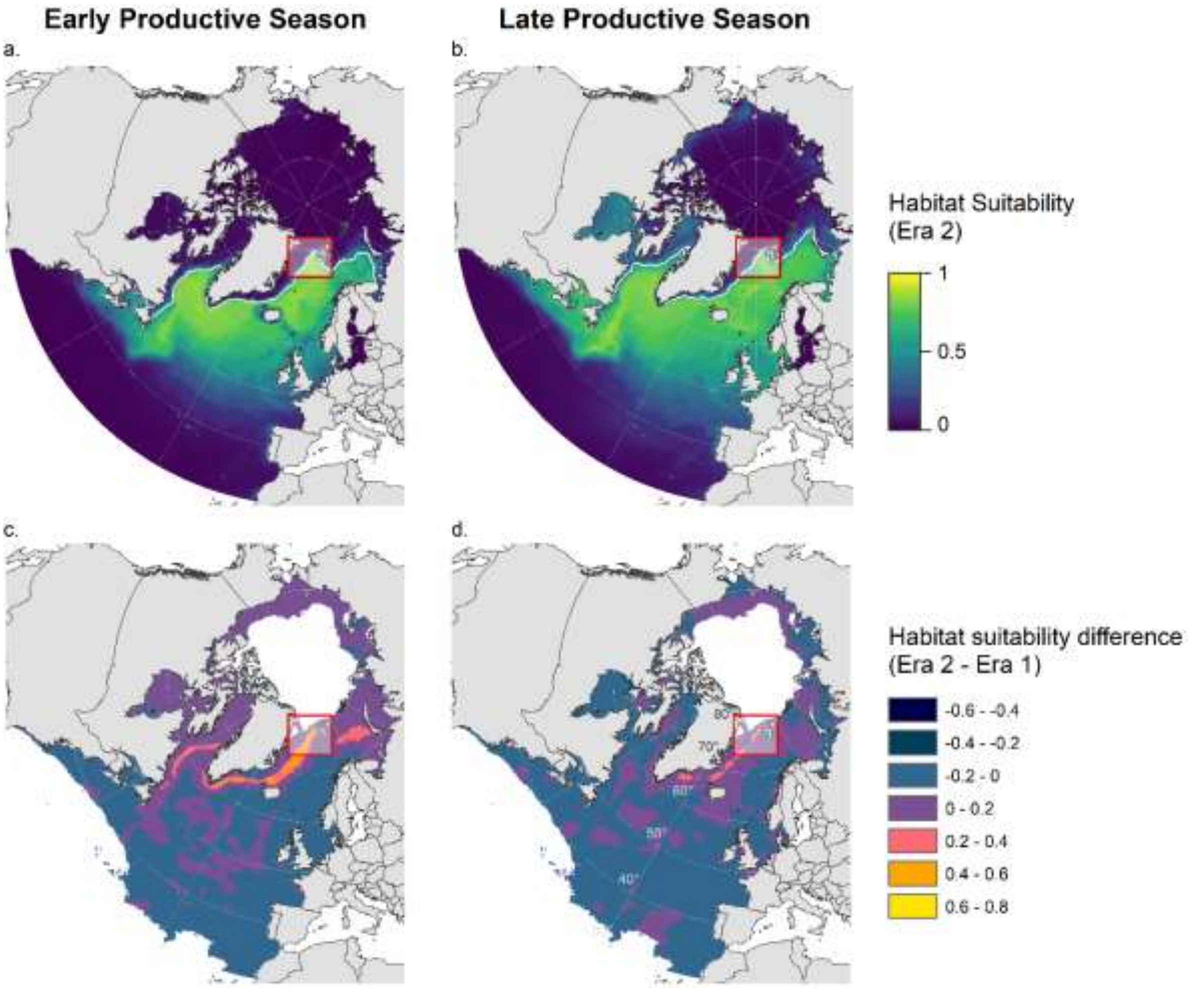

Figure S3: Gridded habitat suitability for *C. finmarchicus* for each season during a. Early productive season (April-June) and b. Late productive season (July-September), during Era 2 (1985-2017) using MaxEnt's cloglog transformed output. The difference in predicted suitability between Ea 2 and Era 1 for early and late seasons are shown in c-d, respectively. White line denotes the average seasonal position of the sea-ice edge, defined as a sea-ice concentration of 15% and square polygons denotes subarea of focus for this study.

Table S1: List of data sources and characteristics for each environmental predictor variable. Source citations given below.

Variable	Source	Original resolution (degrees)	Unit	URL to data source	Availability Era 1	Availability Era 2
Temperature	WOA18[1]	0.25	°C	https://www.nodc.noaa.gov/OC5/woa18/woa18data.html	55-84	85-17
Salinity	WOA18[2]	0.25	psu	https://www.nodc.noaa.gov/OC5/woa18/woa18data.html	55-84	85-17
Sea-ice concentration	NSIDC[3]	0.25	%	https://nsidc.org/data/g10010	55-84	85-13
Current velocity	ORAS-4[4]	1	m.s-1	http://icdc.cen.uni-hamburg.de/las/getUI.do?dsid=id-0d88015986&catid=20B1C0C992F03B0505A7B7A159FCE818&varid=so-id-0d88015986&plot=XY_zoomable_image&view=xy&auto=true	55-84	85-17
Silicate	PISCES[5]	0.25	µmol.kg-1	http://marine.copernicus.eu/services-portfolio/access-to-products/?option=com_csw&view=details&product_id=GLOBAL_REANALYSIS_BIO_001_029	NA	93-17
Chlorophyll a	PISCES[5]	0.25	mg.m-3	http://marine.copernicus.eu/services-portfolio/access-to-products/?option=com_csw&view=details&product_id=GLOBAL_REANALYSIS_BIO_001_029	NA	93-17
pH	PISCES[5]	0.25	total scale	http://marine.copernicus.eu/services-portfolio/access-to-products/?option=com_csw&view=details&product_id=GLOBAL_REANALYSIS_BIO_001_029	NA	93-17
Irradiance (PAR)	modisPAR[6]	0.08	einstein.m-2.day-1	https://oceancolor.gsfc.nasa.gov/l3/	NA	2002-2017
Bathymetry	STRM_30[7]	0.0083	m	https://topex.ucsd.edu/WWW_html/srtm30_plus.html	-	-
Slope	STRM_30	0.25	degrees	https://topex.ucsd.edu/WWW_html/srtm30_plus.html	-	-

[1] Locarnini, R. A. et al. World Ocean Atlas 2018, Volume 1: Temperature, in NOAA Atlas NESDIS 81, A. Mishonov, Ed. (Silver Spring, 2019) pp. 1-52.
[2] Zweng, M. M. et al. World Ocean Atlas 2018, Volume 2: Salinity, in NOAA Atlas NESDIS 81, A. Mishonov, Ed. (Silver Spring, 2019) pp. 1-50.
[3] Walsh, J. E. et al. (2019) Gridded Monthly Sea Ice Extent and Concentration, 1850 Onward, Version 1.1. Boulder, Colorado USA. NSIDC: National Snow and Ice Data Center.
[4] Balmaseda, M. A. et al. (2013) Evaluation of the ECMWF ocean reanalysis system ORAS4. Quarterly Journal of the Royal Meteorological Society, 139: 1132-1161.
[5] Global Monitoring and Forecasting Center (2019) Global Ocean Biogeochemsitry Hindcast Product, E.U. Copernicus Marine Service Information. Available at: https://resources.marine.copernicus.eu/?option=com_csw&view=details&product_id=GLOBAL_REANALYSIS_BIO_001_029

[6] NASA Goddard Space Flight Center, Ocean Ecology Laboratory, Ocean Biology Processing Group. Moderate-resolution Imaging Spectroradiometer (MODIS) Aqua Photosynthetically Available Radiation Data; 2018 Reprocessing. NASA OB.DAAC, Greenbelt, MD, USA.
[7]Becker, J. J., et al. (2009) Global bathymetry and elevation data at 30 arc seconds resolution: SRTM30_PLUS. Marine Geodesy, 32: 355-371.
